# Local failure after definitive radiation treatment of lymph-node positive prostate cancer: supporting the use of novel imaging techniques to personalize treatment options

**DOI:** 10.1259/bjrcr.20200001

**Published:** 2020-04-17

**Authors:** Avinash R Chaurasia, Clayton P Smith, Peter Pinto, Bradford Wood, Erica Schott, Theresa Cooley-Zgela, Ravi Madan, Liza Lindenberg, Esther Mena, Peter Choyke, Deborah Citrin, Baris Turkbey

**Affiliations:** 1 Radiation Oncology Branch, National Cancer Institute, National Institutes of Health, Bethesda, Maryland, USA; 2 Georgetown University School of Medicine, Washington, DC, USA; 3 Urologic Oncology Branch, National Cancer Institute, National Institutes of Health, Bethesda, Maryland, USA; 4 Center for Interventional Oncology, National Cancer Institute, National Institutes of Health, Bethesda, Maryland, USA; 5 Genitourinary Malignancies Branch, National Cancer Institute, National Institutes of Health, Bethesda, Maryland, USA; 6 Molecular Imaging Program, National Cancer Institute, National Institutes of Health, Bethesda, Maryland, USA

## Abstract

Patients with lymph-node positive prostate cancer are often treated with external beam radiotherapy with androgen deprivation therapy^1^, but are expected to have a high rate of biochemical failure. Recently, MRI and molecular imaging have afforded the opportunity to elucidate otherwise occult sites of recurrence after conventional imaging. We present an unusual case of local failure within the prostate after definitive radiation treatment of lymph-node positive prostate cancer, in which advanced imaging allowed for a potentially curative salvage treatment option.

## Clinical presentation

A 55-year-old male first presented in May of 2013 with an elevated serum prostate-specific antigen (PSA) of 21.97 ng dl^−1^ with no palpable prostate nodule on digital rectal examination. An MRI of the prostate revealed a 1.9 cm right apical-mid anterior transition zone lesion suspicious for malignancy (Overall PI-RADS 5). Subsequent MRI transrectal ultrasound fusion-guided biopsy of the prostate was performed with 12 systematic cores and six additional targeted cores. Nine of 18 cores (three of 12 systemic cores, six of six targeted cores, all right-sided) contained Gleason 4 + 4=8 disease. A CT scan of the abdomen and pelvis revealed right pelvic sidewall adenopathy with the largest lymph node measuring 1.7 cm. A CT-guided right pelvic lymph-node biopsy confirmed metastatic prostate cancer. The patient underwent definitive external beam radiation therapy with neoadjuvant, concurrent, and adjuvant androgen deprivation therapy (ADT) with a combination of bicalutamide and leuprolide. Radiotherapy included treatment to the prostate and proximal seminal vesicles to a dose of 79.2 Gy, pelvic lymph nodes to 66.6 Gy, and elective pelvic lymph nodes and full seminal vesicles to 45 Gy, all treated sequentially in 1.8 Gy daily fractions (total of 44 daily fractions). The patient opted to discontinue ADT after 18 months of adjuvant therapy (two years of total ADT). The patient had mild acute and late toxicity, limited to CTCAE Grade one urinary frequency and urgency. Upon testosterone recovery, the PSA rose, meeting criteria for biochemical failure at 4 years after completion of radiation and 29 months after completion of ADT (PSA 2.04 ng dl^−1^, PSA doubling time of 6 months).

## Investigations/Imaging Findings

**Figure 1.**
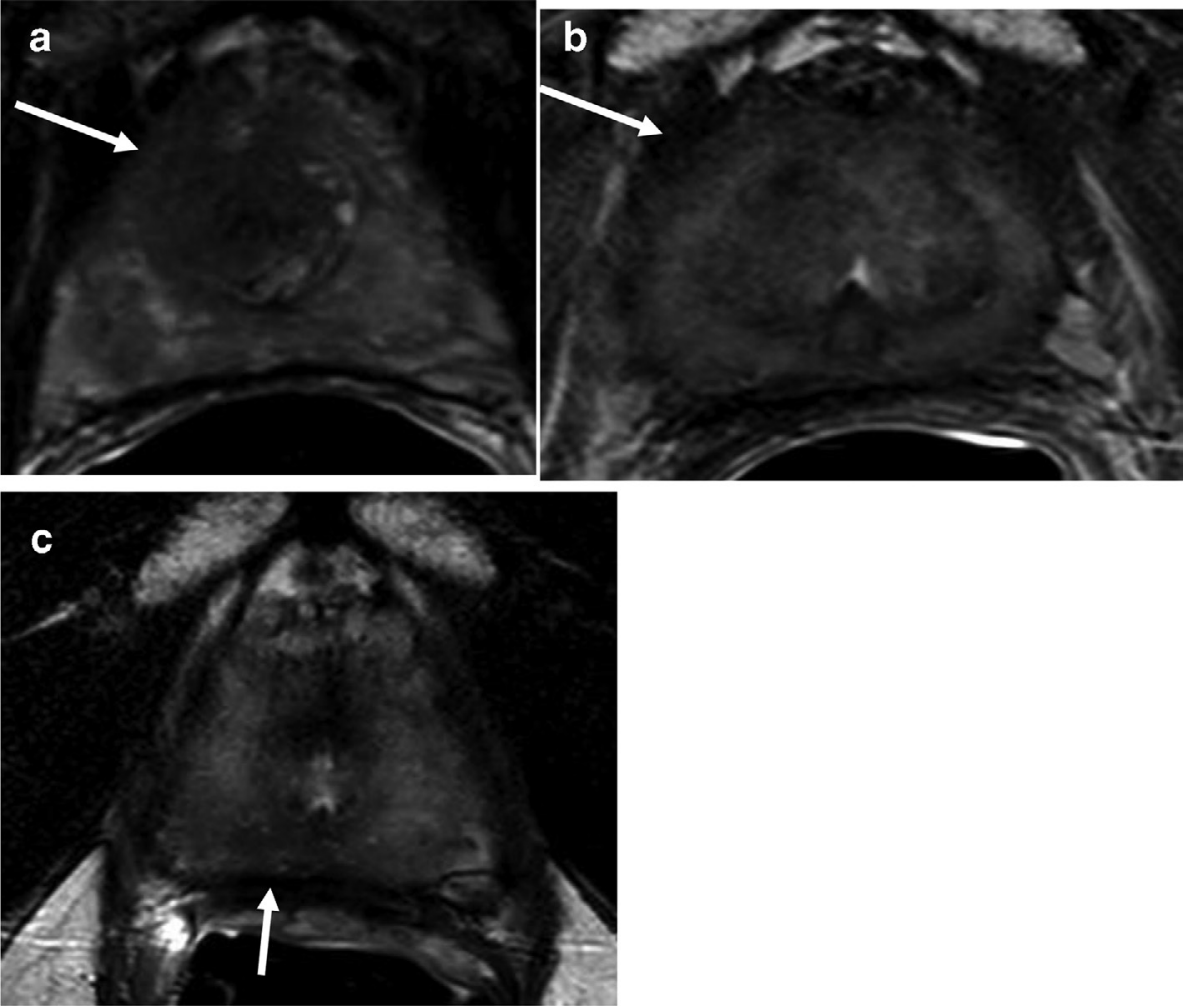
Axial T2W MRI shows an ill defined lesion affecting the right apical-mid anterior peripheral and transition zones prior to treatment (A). Post-treatment axial T2W MRI shows diffuse iso-hypointense signal pattern secondary to treatment effects, with findings consistent with treatment response and low suspicion of residual disease (B). Axial T2W MRI after biochemical recurrence shows diffuse iso-hypointense signal pattern secondary to treatment effects along with a hypointense lesion in the midline to right apical peripheral zone, suspicious for recurrent prostate cancer (C).

**Figure 2.**
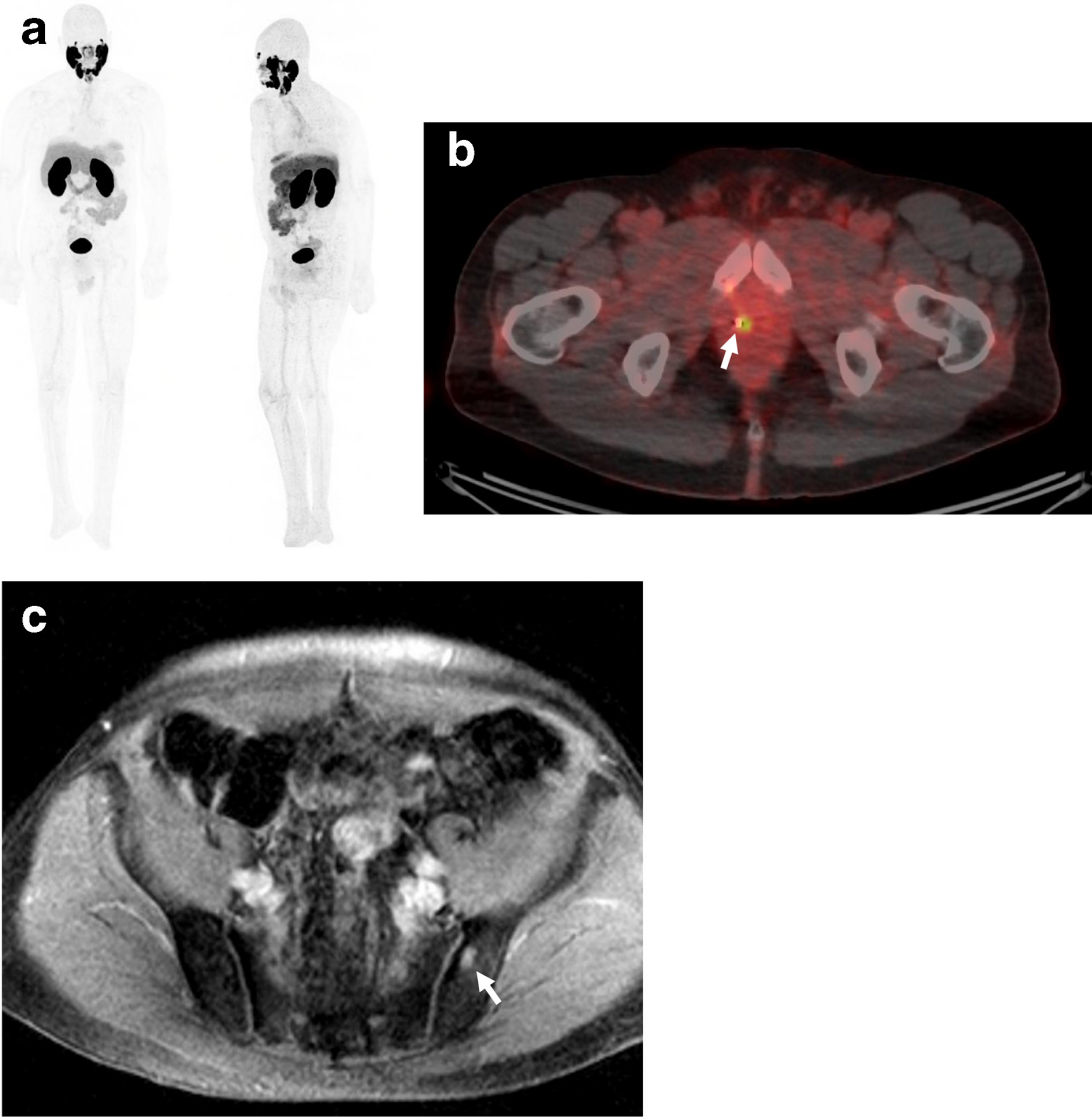
Whole body Prostate Specific Membrane Antigen Positron Emission Tomography (PSMA PET/CT, A), and axial slices of PSMA PET/CT (B) shows focal tracer uptake within the midline to right apical peripheral zone, without uptake in any previously defined prostate lesions, lymph nodes, or bone. MRI of the pelvis/prostate showed indeterminate area in the left ilium (C), without a correlate found on PSMA PET/CT or NaF PET/CT (not pictured).

At the time of biochemical recurrence, the patient underwent imaging and re-staging evaluation. A CT scan of the abdomen and pelvis did not demonstrate the previously seen pelvic sidewall adenopathy or other evidence of recurrence and a nuclear medicine bone scan did not demonstrate specific evidence of osseous metastatic disease. An MRI of the prostate demonstrated resolution of the original right apical-mid anterior transition zone lesion, and emergence of a previously unobserved midline apical-mid peripheral zone PIRADS four lesion (Figure 1). Also noted on this MRI was a suspicious enhancing lesion in the left ilium (Figure 2), which when compared to baseline imaging was felt to represent a benign process that became more prominent due to radiotherapy-related bone marrow changes, mainly associated with fatty replacement. On retrospective image registration with previous radiation plan, both the resolved right apical-mid anterior transition zone lesion and the new suspicious midline apical-mid peripheral zone lesion received the full prescribed radiation dose of 79.2 Gy.

The patient had additional investigational imaging to help clarify if there was distant spread of disease. A prostate-specific membrane antigen (PSMA)-targeted positron emission tomography (PET), PET/CT was performed ([^18^F]DCF-PyL), which revealed an area of uptake in the midline apical peripheral zone concordant with MRI findings, but without evidence of any avid lymph nodes or bone lesions (Figure 2). A sodium fluoride PET/CT (^18^F-NaF PET/CT) scan was performed to clarify the nature of the left iliac lesion, and demonstrated mild focal uptake, suspicious but indeterminate. ^18^F-NaF PET/CT also showed evidence of a new focal uptake in a small left anterior sclerotic iliac bone lesion that was not seen on the MRI scan, but that in retrospect had been present on staging CT scan in 2013.

An extensive multidisciplinary discussion led to the conclusion that the identified bone lesions were less likely to be related to metastatic disease based on imaging characteristics. The decision was made to pursue biopsy of both the iliac lesions seen on the MRI and ^18^F-NaF PET scan. Biopsies of both lesions showed cortical and trabecular bone and no evidence of metastatic prostate cancer.

Given the lack of evidence of metastatic disease, it was felt that a post-radiation MRI-guided TRUS prostate biopsy was appropriate to define the location of recurrence and to clarify treatment options. Pathology from this biopsy revealed 12 systematic cores with no viable malignancy and treatment effect, as well as two targeted cores from the midline apical-mid peripheral zone demonstrating Gleason 4 + 4=8 disease.

## Treatment

The patient was evaluated in a prostate multidisciplinary clinic where he met with urological oncology, radiation oncology, and medical oncology to discuss various treatment options. Ultimately, the patient decided to pursue surgical salvage therapy for his presumed localized recurrence with a robotic-assisted laparoscopic radical prostatectomy with bilateral extended pelvic lymph node dissection that included bilateral external iliac, internal iliac, and obturator lymph nodes.

## Outcome and Follow-up

Surgical pathology after salvage radical prostatectomy revealed Gleason 4 + 4=8 disease in a single focal region of the prostate occupying 5% of the prostate volume in the midline apical-mid peripheral zone lesion, corresponding to pre-operative MRI findings of new suspicious lesion. The remainder of the gland shows extensive post-radiation treatment effect, including the initial right apical-mid anterior transition zone lesion treated with radiation. There were negative margins, no extraprostatic extension, and no seminal vesicle invasion. There were 18 total lymph pelvic nodes (including bilateral obturator and bilateral iliac lymph nodes) removed without any evidence of metastases. The patient recovered well post-operatively and his post-operative PSA is undetectable (<0.02 ng ml^−1^) at 19-month follow-up. Acute toxicity was limited to CTCAE Grade two urinary incontinence and Grade two urinary frequency. Late toxicity up to 18-month follow-up was limited to CTCAE Grade 1 to Grade 2 urinary incontinence and Grade two urinary frequency. No significant acute or late gastrointestinal toxicity was noted.

## Discussion

While previous retrospective studies have shown local failure after treatment of high-risk prostate cancer patients, our case appears to be one of the first mentioned in the literature where the patient had pathologically confirmed lymph-node positive disease at diagnosis and then recurred locally within the prostate without evidence of distant metastasis. The largest series described in the literature analyzed anatomic patterns of recurrence, and of those with an isolated first-recurrence site (versus multiple sites of recurrence), high-risk patients demonstrated 12.2% rate of local failure, 3.3% rate of pelvic lymph node failure, and 9.8% rate of failure in the bone. They suggested that while it has been accepted that high-risk patients are at a higher risk for distant metastasis following biochemical recurrence, they also found a higher absolute risk for isolated local recurrence. They suggest that these patients could benefit from further prostate imaging and post-treatment biopsy.^2^ Existing literature does not help to delineate what the typical failure pattern is for high-risk prostate cancer patients. However, it appears that the more specific and novel imaging studies, PSMA targeting PET/CT and ^18^F-NaF PET/CT scans, were able to more accurately localize the patient’s recurrence to a local site. With the combination of modern dose-escalated IMRT in conjunction with such advanced imaging, these areas of gross nodal disease can be treated effectively with retrospective data showing excellent rates of biochemical control of 62–83% at 5 years and 63% at 10 years.^3^

PSMA is highly expressed in prostate cancer, and its expression increases with tumor aggressiveness, metastatic disease, and rapid recurrence. However, it is also expressed in normal healthy tissue (*i.e.,* colonic tissue, renal tissue, salivary glands) and non-prostatic malignant tissue (*i.e.,* colon carcinoma, renal cell carcinoma, transitional cell carcinoma),^4^ and this can confound the results found when performing such a scan.^4^Nevertheless, several retrospective series have shown high rates of sensitivity and specificity as well as high detection rates even with low serum PSA below 0.5 ng ml^−1^.^5^ In a retrospective series of 319 patients with biochemical recurrence after primary therapy, ^68^Ga-PSMA-ligand PET/CT demonstrated a lesion-based sensitivity, specificity, negative predictive value (NPV) and positive predictive value (PPV) of 76.6 %, 100 %, 91.4% and 100%, respectively.^6^ Another retrospective analysis of biochemical recurrence after radical prostatectomy found a lesion detection rate of >90% with PSA levels more than 1 ng ml^−1^ and a significantly higher detection rate in patients with Gleason score ≥8 versus ≤7.^7,8^ It has been suggested by some groups that PSMA PET/CT should be the gold standard for imaging in biochemical recurrent prostate cancer as well as having utility in staging and assessing treatment response.^9^

The patient presented herein represents an anecdotal case of biochemical recurrence after radiation therapy and ADT, a situation in which PSMA targeting PET/CT is not described well in the literature. In this patient, the use of conventional and novel imaging modalities as well as confirmatory biopsy guided treatment. There was significant discordance between expected pattern of failure and conventional imaging findings and with the help of more sensitive advanced imaging, radiographical evidence of metastatic disease was excluded. While there may have been occult disease not detectable based on the described imaging techniques, our multidisciplinary discussion led to the aforementioned treatment plan.

^18^F-NaF PET/CT has more favorable pharmacokinetics, better image quality, higher uptake in bone, and shorter imaging time than conventional technetium-99m-MDP bone scan (Tc-99m) for bone metastases; however, its uptake is non-specific compared to other PET studies. Its uptake reflects osteoblastic activity, which is not necessarily specific to malignancy (*i.e.,* degenerative disease, old or healing fractures, other benign conditions). Early studies show that it can allow for detection of lytic and sclerotic metastases and may also allow for detection of occult bone metastases, with positive results occurring at lower serum PSA levels than with Tc-99m.^10^ Various early studies of sensitivity and specificity of NaF PET/CT with relatively high sensitivities of around 81–93% but varying specificities of 54–93%.^10^ The initial results of the National Oncologic PET Registry found that it allowed for personalized treatment decisions that revised previous treatment plans in 40% of those patients.^11^ For our patient, there were two indeterminate lesions, both of which were biopsy negative. While we cannot rule out the possibility of a false-negative biopsy, the absence of uptake on PSMA-based imaging coupled with a negative biopsy increases the confidence that this incidental finding is benign. In this case, NaF PET/CT did change the post-imaging evaluation plan.

In conclusion, investigational imaging studies discussed above show great promise at discovering occult locations of recurrence with heightened sensitivity, which can guide personalized decision making for oncological care. The use of multiple advanced imaging modalities in complex cases, such as biochemically recurrent prostate cancer with unclear location of recurrence, can also serve as tools to provide more data to clinicians completing staging and making critical decisions regarding next best steps in management. As these modalities become more prevalent in the community, there is a growing opportunity for personalization of treatment approach in complex clinical scenarios.

## Learning points

1. Advanced imaging techniques such as prostate-specific membrane antigen targeting PET/CT and ^18^F-NaF PET/CT may detect occult disease earlier than conventional imaging.
2. Advanced imaging techniques have higher sensitivity compared to conventional imaging, and both generations of imaging can be used in a multidisciplinary setting to personalize treatment decisions.
3. Aggressive local therapy can sterilize lymph-node positive high-risk prostate cancer even in the recurrence setting, and these cases require complex personalized clinical decisions.

## References

[1] James ND, Spears MR, Clarke NW, Dearnaley DP, Mason MD, Parker CC, et al Failure-Free survival and radiotherapy in patients with newly diagnosed nonmetastatic prostate cancer: data from patients in the control arm of the STAMPEDE trial. JAMA Oncol 2016; 2: 348–57. doi: 10.1001/jamaoncol.2015.4350 26606329 PMC4789485

[2] Zumsteg ZS, Spratt DE, Romesser PB, Pei X, Zhang Z, Kollmeier M, et al Anatomical patterns of recurrence following biochemical relapse in the dose escalation era of external beam radiotherapy for prostate cancer. J Urol 2015; 194: 1624–30. doi: 10.1016/j.juro.2015.06.100 26165583 PMC5003416

[3] Créhange G, Chen CP, Hsu CC, Kased N, Coakley FV, Kurhanewicz J, et al Management of prostate cancer patients with lymph node involvement: a rapidly evolving paradigm. Cancer Treat Rev 2012; 38: 956–67. doi: 10.1016/j.ctrv.2012.05.005 22703831 PMC3739983

[4] Silver DA, Pellicer I, Fair WR, Heston WD, Cordon-Cardo C Prostate-Specific membrane antigen expression in normal and malignant human tissues. Clin Cancer Res 1997; 3: 81–5. 9815541

[5] Bouchelouche K, Turkbey B, Choyke PL Psma PET and radionuclide therapy in prostate cancer. Semin Nucl Med 2016; 46: 522–35. doi: 10.1053/j.semnuclmed.2016.07.006 27825432 PMC5123597

[6] Afshar-Oromieh A, Avtzi E, Giesel FL, Holland-Letz T, Linhart HG, Eder M, et al The diagnostic value of PET/CT imaging with the (68)Ga-labelled PSMA ligand HBED-CC in the diagnosis of recurrent prostate cancer. Eur J Nucl Med Mol Imaging 2015; 42: 197–209. doi: 10.1007/s00259-014-2949-6 25411132 PMC4315487

[7] Eiber M, Maurer T, Souvatzoglou M, Beer AJ, Ruffani A, Haller B, et al Evaluation of hybrid 68Ga-PSMA ligand PET/CT in 248 patients with biochemical recurrence after radical prostatectomy. J Nucl Med 2015; 56: 668–74 05. doi: 10.2967/jnumed.115.154153 25791990

[8] Rowe SP, Gorin MA, Allaf ME, Pienta KJ, Tran PT, Pomper MG, et al Pet imaging of prostate-specific membrane antigen in prostate cancer: current state of the art and future challenges. Prostate Cancer Prostatic Dis 2016; 19: 223–30 09. doi: 10.1038/pcan.2016.13 27136743 PMC4982789

[9] Zschaeck S, Lohaus F, Beck M, Habl G, Kroeze S, Zamboglou C, et al PSMA-PET based radiotherapy: a review of initial experiences, survey on current practice and future perspectives. Radiat Oncol 2018; 13: 90. doi: 10.1186/s13014-018-1047-5 29751842 PMC5948793

[10] Wallitt KL, Khan SR, Dubash S, Tam HH, Khan S, Barwick TD Clinical PET imaging in prostate cancer. Radiographics 2017; 37: 1512–36 Sep-Oct. doi: 10.1148/rg.2017170035 28800286

[11] Hillner BE, Siegel BA, Hanna L, Duan F, Shields AF, Quinn B, et al Impact of (18)F-Fluoride PET on Intended Management of Patients with Cancers Other Than Prostate Cancer: Results from the National Oncologic PET Registry. J Nucl Med 2014; 55: 1054–61. doi: 10.2967/jnumed.113.135475 24819422

